# HMG-CoA Lyase Deficiency: A Retrospective Study of 62 Saudi Patients

**DOI:** 10.3389/fgene.2022.880464

**Published:** 2022-05-13

**Authors:** Majid Alfadhel, Basma Abadel, Hind Almaghthawi, Muhammad Umair, Zuhair Rahbeeni, Eissa Faqeih, Mohammed Almannai, Ali Alasmari, Mohammed Saleh, Wafaa Eyaid, Ahmed Alfares, Fuad Al Mutairi

**Affiliations:** ^1^ Genetics and Precision Medicine Department, King Abdullah Specialized Children Hospital, King Abdulaziz Medical City MNG-HA, Riyadh, Saudi Arabia; ^2^ Medical Genomics Research Department, King Abdullah International Medical Research Center (KAIMRC), King Saud Bin Abdulaziz University for Health Sciences, Ministry of National Guard Health Affairs (MNG-HA), Riyadh, Saudi Arabia; ^3^ Medical Genetics Department, King Faisal Specialist Hospital and Research Centre, Riyadh, Saudi Arabia; ^4^ Section of Medical Genetics, Children’s Specialist Hospital, King Fahad Medical City, Riyadh, Saudi Arabia; ^5^ Department of Pediatrics, College of Medicine, Qassim University, Qassim, Saudi Arabia; ^6^ Division of Translational Pathology, Department of Laboratory Medicine, King Abdulaziz Medical City, Riyadh, Saudi Arabia

**Keywords:** Ketogenesis, hypoglycemia, HMG-CoA lyase, Saudi Arabia, ketone body synthesis, acidosis, HMGCL

## Abstract

3-Hydroxy-3-methylglutaryl-coenzyme A lyase deficiency (HMG-CoA lyase) is a rare inborn error of leucine degradation and ketone body synthesis, caused by homozygous or compound heterozygous disease-causing variants in *HMGCL*. To understand the natural history of this disease, we reviewed the biochemical, clinical, and molecular data of 62 patients from 54 different families with confirmed HMG-CoA lyase deficiency (HMGCLD) diagnosis from Saudi Arabia. The majority of the affected individuals were symptomatic. At initial diagnosis, 38 patients (61.29%) presented with hypoglycemia and 49 patients (79.03%) developed metabolic acidosis. In 27 patients (43.54%), the disorder manifested in the neonatal period, mostly within the first days of life, while 35 (56.45%) patients were diagnosed within the first year of life or beyond. All the patients were alive and developed long-term neurological complications during data collection, which may significantly influence their quality of life. Common neurological findings include seizures 17/62 (27.41%), hypotonic 3/62 (4.83%), speech delay 7/62 (11.29%), hyperactivity 4/62 (4.83%), developmental delay 6/62 (9.677%), learning disability 15/62 (24.14%), and ataxic gate 1/62 (1.612%). An MRI of the brain exhibited nonspecific periventricular and deep white matter hyperintense signal changes in 16 patients (25.80%) and cerebral atrophy was found in one (1/62; 1.612%) patient. We identified a founder variant [c.122G>A; p.(Arg41Gln)] in 48 affected individuals (77.41%) in the *HMGCL* gene. This is the largest cohort of HMGCLD patients reported from Saudi Arabia, signifying this disorder as a likely life-threatening disease, with a high prevalence in the region. Our findings suggest that diagnosis at an early stage with careful dietary management may avoid metabolic crises.

## Introduction

**FIGURE 1:**
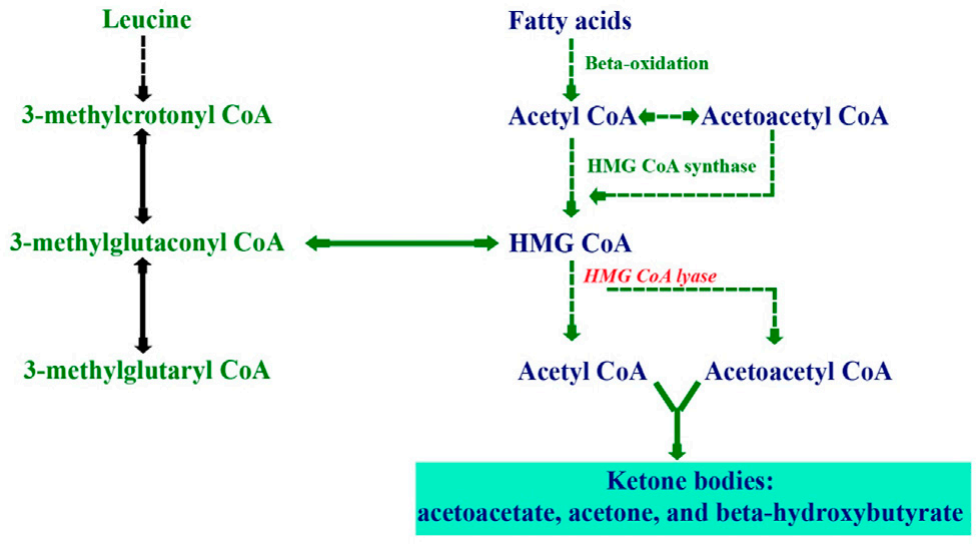
Biochemical pathways illustrating ketogenesis through the metabolism of fatty acids and leucine.

3-Hydroxy-3-methylglutaryl-CoA lyase deficiency, also known as HMG-CoA lyase deficiency (HMGCLD; OMIM 246450), is a rare inherited autosomal-recessive metabolic disorder caused by disease-causing homozygous or compound heterozygous variants in the *HMGCL*. This gene is located in the short arm of chromosome 1p36.11 (Pié et al., 2007; Menao et al., 2009), which encodes the enzyme 3-hydroxymethyl-3-methylglutaryl-coenzyme A lyase (HMG-CoA lyase). This mitochondrial enzyme has a role in dietary protein metabolism, which forms acetyl-CoA and acetoacetate from HMG-CoA, known as the final step of leucine degradation and fat breakdown for energy (Puisac et al., 2010; Václavík et al., 2020) (Figure 1). Therefore, the body cannot synthesize ketone bodies, which are essential for energy production during the fasting period (Václavík et al., 2020). The prevalence of this disorder is less than 1/100,000 in live borns (Santarelli et al., 2013), while in Saudi Arabia, the incidence is 1: 55,357 (Alfadhel et al., 2017) which is approximately twice the world incidence.

HMGCLD clinically manifests early during the neonatal period (Santarelli et al., 2013) or later within the first year of life. Acute presentations include seizures, vomiting, metabolic acidosis, lethargy, and hypoketotic hypoglycemia (Pié et al., 2007; Menao et al., 2009). These symptoms sometimes progress to coma and death (Robinson, 2006).

HMGCLD can be diagnosed through newborn screening by measuring 3-hydroxy isovaleryl carnitine (C5-OH) via the tandem mass spectrometry methodology, which is usually elevated in this disease, and subsequently, the diagnosis is confirmed by the measurement of organic acids which showed a characteristic pattern of increased 3-methylglutaric, 3-hydroxy-3-methylglutaric, and 3-hydroxyisovaleric acids. In addition, urinary levels of 3-methyl-crotonyl glycine may also be increased (Alfadhel et al., 2017).

Also, it can be detected by low enzymatic activity testing using lymphocytes, fibroblasts, or immortalized lymphoblastoid cells. The molecular diagnosis is achieved if it shows homozygous or compound heterozygous pathogenic variants in the *HMGCL* gene using Sanger sequencing. Despite the high incidence in Saudi Arabia, a few studies are associated with natural history, epidemiological, clinical, and molecular data related to HMGCLD pathogenesis.

In the present study, we report the largest cohort of HMGCLD from Saudi Arabia. In addition, we provide clinical and molecular data associated with HMG-CoA lyase deficiency. Also, we provided the psychological evaluation of some patients to give an insight into long-term outcomes.

## Materials and Methods

### Ethical Approval

The Institutional Review Board (IRB) of King Abdullah International Medical Research Centre (KAIMRC), Riyadh (Ref. RC19/120/R), approved the study. Written informed consent for the publication of genetic, molecular, and clinical data was obtained from the parents of the enrolled patients.

### Study Subjects

Individuals with HMGCLD were recruited between 1987 and 2018 from Riyadh, Saudi Arabia. A total of 62 patients were included in the study, recruited from three multicenters in Riyadh, the Kingdom of Saudi Arabia (KSA).

### Inclusion Criteria

Inclusion criteria encompassed those individuals having confirmed molecular diagnoses. The data were collected to extrapolate the natural history, clinical presentation, biochemical and molecular results, follow-up assessment of neurological status, complications, and treatment compliance for all the enrolled affected individuals.

### Clinical Information and Growth Parameters

Detailed clinical data were collected from the patient charts of all affected individuals from the respective hospitals. The concerned clinicians provided the patient data such as age, weight, height, and developmental milestones. The provided data were changed to percentiles to standardize measurements. Data regarding neonatal diagnosis, consanguinity, biochemical tests, developmental delay, and other associated features were also provided (Table 1).

**TABLE 1:** Summary of the 62 patients investigated in the present study compared to already reported cases in the literature.

Studies	Present study Alfadhel et al. ( 2021)	Grunert et al. (2017)	Menao et al. (2009)	Boutouchent et al. (2021)	Bischof et al. (2004)	Reimão et al. (2009)
Total patients	62	37	24	1	1	1
Clinically investigated	62	34/37 (91.89%)	24	1	1	1
Females	31/62 (50%)	20/37 (54.05%)	N/A	+	+	-
Males	31/62 (50%)	17/37 (45.94%)	N/A	-	-	+
Diagnosed in the first year of life	35/62 (56.45%)	32/37 (86%)	14/24 (56%)	- (54 years)	- (36 years)	- (29 years)
Consanguinity	62/62 (100%)	16/32 (50%)	N/A	-	-	-
Biochemical investigations						
Positive familial hypercholesterolemia (FH)	31/62 (50%)	N/A	N/A	+	+	+
NBS positive	60/62 (96.77%)	N/A	N/A	-	N/A	N/A
Urine OA	62/62 (100%)	N/A	14/24 (56%)	+	+	N/A
High ammonia	5/62 (8.064%)	19/20 (95%)	N/A	+	+	+
Acidosis	49/62 (79.03%)	20/20 (100%)	24/24 (100%)	-	+	+
Elevated leucine level	50/62 (80.64%)	23/35 (66%)	N/A	+	+	+
Liver impairment	10/62 (16.129%)	N/A	N/A	-	N/A	+
Hypoglycemia	38/62 (61.29%)	25/28 (89%)	Most patients	-	N/A	-
Microcephaly	4/62 (6.45%)	1/34 (3%)	N/A	-	N/A	N/A
Tricuspid insufficiency	1/62 (1.612%)	N/A	N/A	-	N/A	N/A
Clinical investigation						
Microcephaly	4/62 (6.45%)	1/34 (3%)	N/A	-	N/A	N/A
Seizures	17/62 (27.41%)	17/34 (50%)	N/A	+	+	+
Hypotonia	3/62 (4.83%)	11/34 (32%)	5/24 (20.88%)	+	N/A	N/A
Ataxic gate	1/62 (1.612%)	2/34 (6%)	N/A	+	+	+
Spasticity	3/62 (4.83%)	1/34 (3%)	N/A	N/A	N/A	N/A
Developmental delay	6/62 (9.677%)	13/35 (37%)	N/A	-	-	-
Hyperactivity	4/62 (6.45%)	15/34 (44.11%)	N/A	+	N/A	-
Speech delay	7/62 (11.29%)	16/35 (46%)	N/A	-	-	-
Learning disability	15/62 (24.14%)	17/34 (50%)	N/A	-	-	-
Hepatomegaly	12/62 (19.35%)	14/34 (41%)	5/24	-	-	+
Fatty liver	10/62 (16.12%)	N/A	N/A	+	+	+
High liver enzyme	9/62 (14.51%)	26/37 (70%)	3/24 (12.5%)	-	-	+
Gall stone	4/62 (6.45%)	N/A	N/A	-	-	-
Pancreatitis	1/62 (1.612%)	-	N/A	N/A	-	N/A
Hydronephrosis	1/62 (1.612%)	N/A	N/A	-	-	N/A
Vesicouretric reflux	1/62 (1.612%)	N/A	N/A	-	-	N/A
Reye-like syndrome	1/62 (1.612%)	N/A	N/A	-	-	N/A
Molecular investigation						
Available genetic test	55/62 (88.70%)	34/37 (91.89%)	24/24 (100%)	1	1	N/A
Most frequent variant in the *HMGCL* gene	c.122G > A p.(Arg41Gln) 48/62 (77.41%)	c.876+1G > C 10/37 (27.02%)	c.109G > T; p.(Glu37*) 10/24 (41.66%)	c.144G > C; p.(Lys48Asn)	N/A	N/A
Treatment						
Formula (I-valex special formula)	14/62 (22.58%)	23/35 (66%)	N/A	-	-	N/A
Carnitine supplement	17/62 (27.41%)	31/34 (91%)	N/A	-	-	N/A

### Biochemical Investigations

All patients underwent measurements of the acylcarnitine profile by tandem mass spectrometry and urine organic acids by the gas chromatography method (GC/MS).

### Genetic Testing

DNA molecular testing of the *HMGCL* gene was performed for all patients. In addition, expert medical geneticists in different tertiary centers evaluated the affected individuals with pathogenic, likely pathogenic, or variant of uncertain significance (VUS). Whole exome sequencing (WES) and Sanger sequencing were performed as previously described (Umair et al., 2021; Nashabat et al., 2019; Alghamdi et al., 2020). The clinical laboratory that issued the VUS original report was re-contacted at the time of revision. The identified variants were screened using an in-house exome/genome database containing more than 1,500 control sample data [https://kgd.kaimrc.med.sa/welcome/]. The pathogenicity of the identified variants was checked using different online available tools such as Varsome, MutationTaster, SIFT, Mutation assessor, and PROVEAN.

## Results

### Demographics

The present study includes a retrospective chart review of 62 patients from 54 different Saudi families with confirmed HMGCLD diagnoses. The patients were diagnosed at three centers in Riyadh, KSA. There was equal gender distribution [males: 31 (50%), females: 31 (50%)] (Table 1). All patients were alive at the time of the last follow-up with an age range from 27 months to 32 years.

Thirty-seven patients in these centers are living in the capital city of Riyadh, KSA. Other patients belong to different regions in the KSA, including nine patients from the southern part, nine from the western, two from the eastern, two from the northern region, and four from the northwest area. Consanguinity was observed in all of the 54 families included (100%). A positive family disease history was observed in 31 patients, of which 27 patients had a history of an affected sibling (Table 1).

### Initial Diagnosis

The patients included in this study showed variable clinical presentations that sometimes delay the initial diagnosis. The period of the first clinical presentation is variable among patients. Twenty-seven patients (43.54%) were diagnosed early during the neonatal period of life ranging from the 2nd to 10th day of life, or within 1 month of age, whereas 35 patients (56.45%) were diagnosed later in the infantile period. While two of them were diagnosed during the second year of life, three patients presented in the childhood period and, finally, one at the age of 12 years.

### Clinical Variability and Severity

There were substantial differences in the clinical phenotypic presentation of all the patients. Thirty-eight patients (61.29%) developed hypoketotic hypoglycemia at initial diagnosis, and 49 patients (79.03%) developed metabolic acidosis, presented with respiratory distress, poor feeding, vomiting, and seizures (17/62; 27.41%). Forty (64.51%) patients developed both hypoglycemia and acidosis. Two patients showed hyperammonemia, and five developed hypo-activity and lethargies that led to deep coma.

Some of the patients were prone to metabolic decompensation due to illness requiring hospital admissions, and a few had a record of several hospital admissions because of viral diseases. During the clinical follow-up, 49 patients had a normal examination, and their growth parameters lie within the normal centile. Microcephaly was evident in four of the recruited patients (6.45%), while one had microphthalmia (1.6%) and one patient was observed to have repaired cleft lip (1.6%).

### Long-Term Neurodevelopment Complications

All the patients were alive and developed long-term neurological complications at the time of data collection, which may severely affect their quality of life. Common findings include seizures 17/62 (27.41%), hypotonia 3/62 (4.83%), speech delay 7/62 (11.29%), hyperactivity 4/62 (4.83%), developmental delay 6/62 (9.677%), learning disability 15/62 (24.14%), and ataxic gait 1/62 (1.612%). An MRI of the brain exhibited nonspecific periventricular and deep white matter hyperintense signal changes in 16 patients (25.80%) and cerebral atrophy was found in one (1/62; 1.612%) of the investigated patients.

A total of six patients revealed mild to moderate developmental delay and seven patients showed speech problems ranging from delay to unclear pronunciation. Fifteen patients (24.2%) with learning disabilities had poor school performance, three patients revealed hyperactivity (4.83%), and one of the patients (1.6%) showed hyperactive, impulsive, poorly structured, inattentive, on/off aggressive behavior, and intellectual disability. One of the patients having moderate degrees of cognitive delay revealed an intelligence quotient (IQ) of 38 on the Leiter International Performance Scale.

### Biochemical and Radiological Investigations

Laboratory investigations are essential to make a comprehensive diagnosis. Newborn screening elucidates that 3-hydroxy isovaleryl carnitine (C5-OH) is found in high levels in such a disease and it was positive in all 62 patients (100%). Urine organic acid profiles show high excretion of 3-hydroxyisovaleric acid, 3-hydroxyglutaconic acid, and 3-hydroxy-3-methyl glutaconic acid in all the studied patients. The leucine level was normal in 12 patients (19.35%). Thirty-one patients (50%) revealed familial hypercholesterolemia (FH). Fifty patients (80.64%) developed metabolic distress triggered by febrile illness. As this disorder results in ketogenesis defects, 38 patients (61.29%) revealed hypoglycemia during fasting (10 patients (16.1%) revealed abnormal LFT, while others were in the normal range). An ultrasound examination showed hepatomegaly in 12 (19.35%) investigated patients, while a diffuse fatty liver was observed in 10 patients (16.12%) and gallstones were found in four (645%) patients. A high liver enzyme was observed in nine patients (14.51%), while hydronephrosis, vesicoureteric reflux, and Reye-like syndrome were observed in one patient, each 1/62 (1.6%) (Table 1).

### Molecular Analysis

A molecular diagnosis including WES and Sanger sequencing was performed using standard methods. A variant analysis confirmed the diagnosis for 55/62 patients (88.70%) (Table 1). In seven patients, molecular genetic testing was not ordered. In 48 patients (77.41%), we identified the most frequently reported founder variant [c.122G>A; p.(Arg41Gln)] in the *HMGCL* gene. The c.122G>A variant causes substitution that replaces arginine amino acid at position 41 with glutamine amino acid. All parents were confirmed to be heterozygous carriers of this variant via Sanger sequencing. The variant [c.122G>A; p.(Arg41Gln)] causing HMGCLD is the most frequent in the Saudi Arabian population (Mitchell et al., 1998; Al-Sayed et al., 2006; Pie et al., 2007; Alfadhel et al., 2017).

Similarly, we also revealed a previously reported frame-shift variant (c.206_207del; p(Ser69Cysfs*11)) in five affected individuals (8.064%), previously reported frame-shift variant c.914_915delTT (p.(Phe305Tyrfs*10) in two affected individuals (3.225%), and a novel variant c.599T>A; p.(Ile200Asn) in single affected individual (1.612%) (Table 1).

### Dietary Management

Retrospectively, the obtained nutritional data were very heterogeneous. Most patients received either a leucine-restricted diet (60%) or a protein-restricted diet (22.58%; I-Valex special formula), but most of the patients were not in compliance with the diet regimen. In five patients, the diet was stopped and the patients were shifted to a regular diet. There was no apparent difference observed in the outcomes of the patients having a leucine or protein-restricted diet. However, many patients did not comply with the treatment and only 17 patients (27.41%) received carnitine (50–100 mg/kg/day). Two patients did not continue the treatment.

## Discussion

In this study, we retrospectively reviewed clinical, molecular, biochemical, and radiological findings in 62 patients with HGMCLD. The clinical presentation of HGMCLD is highly heterogeneous but it is usually evident in the neonatal and first year of life. Most affected individuals in our study became symptomatic within the first year after birth (Grunert et al., 2017; Václavík et al., 2020; Grünert and Sass, 2020). HMGCLD patients typically present features such as acute metabolic decompensation that may be life-threatening (Grünert and Sass, 2020).

The biochemical findings include acidosis, hypoglycemia, hyperammonemia, increased plasma/serum transaminase activities, and an increased anion gap. As ketogenesis is vital for maintaining glucose homeostasis, some patients might have hypoglycemia with or without metabolic acidosis during the initial episode that might be fatal due to the accumulation of organic acids. Similarly, ketone body accumulation reduces cerebral and peripheral glucose utilization and decreases muscular proteolysis (Evans et al., 2017). Therefore, for patients with HMGCLD who are unable to synthesize ketone bodies, their glucose utilization will be high, and gluconeogenesis will increase to uphold normoglycemia (Evans et al., 2017; Grunert et al., 2017). Hyperammonemia is also commonly reported; however, five (8.064%) patients showed hyperammonemia in the presented cohort, while all had abnormal urine organic acid profiles. Thus, the pathogenesis mechanism underlying hyperammonemia in HMGCLD patients is possibly multifactorial and still not completely understood.

HMGCLD diagnosis can be achieved using metabolite screening. Previous studies suggested that patients display a distinguishing biochemical profile with typical metabolites in the blood acylcarnitine and/or a urinary organic acid pattern (Václavík et al., 2020). Therefore, the first step in the diagnosis is the MS/MS-based acylcarnitine profiling in dried blood spots/plasma and the GC/MS-based organic acid analysis in urine. Then, molecular diagnosis using next-generation sequencing should be followed to pinpoint precisely the genetic cause of the disease (Ismail et al., 2019; Alfadhel et al., 2021). Similarly, targeted newborn screening should also be initiated to eradicate this severe disorder (Alfadhel et al., 2019). Similarly, Reimão et al. described late-onset diseases where a 29-year-old man with no prior medical history presented sudden coma, profound hypoglycemia, hyperammonemia, and metabolic acidosis without ketosis (Reimão et al., 2009). A 36-year-old woman with a late onset of an acute episode of hyperproteinorachia (0.73 g/L), hypoglycemia, and generalized seizures has also been reported (Bischof et al., 2004). Patients showing different levels of severity in the phenotypic presentation highlight clinical heterogeneity of the disorder. Still, it is difficult to establish a proper genotype–phenotype correlation in HMGCLD (Grunert et al., 2017; Alfadhel et al., 2017).

The mechanism of neurological pathophysiology in HGMCLD is still poorly understood; however, possible mechanisms include secondary carnitine deficiency, hypoketotic hypoglycemia, and intracellular toxic organic acid accumulation (Puisac et al., 2010). Furthermore, MRI shows a unique combination of T2-weighted diffuse mild signal changes with multiple foci of a more severe signal abnormality (Legault et al., 2015). Studies using rats revealed that the accumulation of the metabolite in HMGCLD results in oxidative stress in the developing rat’s striatum that disrupts bioenergetics dynamics, signaling pathways, and ER–mitochondria communication, which might explain the HMGCLD disease pathogenesis (da Rosa et al., 2015). Therefore, it might be concluded that these events cause the onset and progression of symptoms during decompensation in the neonatal period (da Rosa et al., 2020).

There have been notable highlights of some of the complications of the disease in recent years as HMCLD causes varying damages to the brain, liver, pancreas, and a very rare heart defect. Seizure is the most common complication in 27.14% (n: 17) of the presented patients. An MRI of the brain exhibited nonspecific periventricular and deep white matter hyperintense signal changes in 16 patients and cerebral atrophy was also found in one patient. Practically the same as the previously described marked lesions lie in periventricular white matter, arcuate fibers, and most prominently in the frontal or periarterial region, and slightly hyperintense subcortical white matter signals were also found (Yalçinkaya et al., 1999). Developmentally affected patients exhibit a delay in motor, speech, adaptive, and communication skills ranging from mild to moderate delay, found in 9.67% (n: 6) to profound intellectual disabilities found in one patient. However, in the present cohort, five patients had normal development despite the same MRI findings as previously described.

As HMGCL-deficient patients are prone to metabolic decompensation during illness, the presented patients demonstrated the same. Out of 37 patients reported previously with HMGCL variants, 10 had no metabolic decompensations after diagnosis, while 22 had at least one metabolic crisis, primarily associated with infections (Grunert et al., 2017). Similarly, one of the present patients had 10 metabolic decompensations, while several others had never had a hospital admission before. This signifies the heterogeneity of the disorder and the involvement of additional vital players defining the pathogenicity of the disease. In addition, about 15% of patients in the study reported by Grunert et al. (2017) died before the time of data collection. However, none of the patients reported in the present study died.

An 8-month-old infant with HMG-CoA lyase deficiency revealed recurrent pulmonary infections, left ventricular noncompaction, macrocephaly, nonketotic hypoglycemia, metabolic acidosis, and seizure (Köksal et al., 2015). However, in the present cohort, left ventricular noncompaction, macrocephaly, and recurrent pulmonary infections were not observed. Similarly, 10 novel *HMGCL* variants were reported in 24 patients of different origins suffering from 3-hydroxy-3-methyl-glutaric aciduria (Menao et al., 2009). The main complications in these patients were hepatic involvement with hepatomegaly and elevation of transaminases, with nine patients showing these symptoms during the neonatal period (Menao et al., 2009). However, in our cohort, 12 patients (19.35%) revealed hepatomegaly, and 27 patients (43.54%) were diagnosed during the neonatal period. Furthermore, Ozand et al. (1991) have reported overlapping clinical presentations, where 11 Saudi infants suffered from HMG-CoA lyase deficiency, with clinical presentation of acidotic attack including hyperpnea, lethargy, seizures, and tachypnea, at either birth or refusal of food in later infancy (Ozand et al., 1991).

Based on our data, no special diet or carnitine supplementation can be suggested to the patients. However, most of the patients were on a protein and/or leucine-restricted diet. Based on clinical reports and patho-biochemical considerations, the avoidance of fasting seems to be the mainstay of therapy for ketogenesis. In addition, administration of l-carnitine may have detoxifying effects and help to avoid secondary l-carnitine deficiency and intracellular depletion of free coenzyme A (Mitchell et al., 2008, Grünert and Sass, 2020).

The molecular study revealed that missense homozygous variants in the *HMGCL* gene 9c.122G>A; p.(Arg41Gln)] were identified in 48 patients (77.41%). Previously, the c.122G>A; p.(Arg41Gln) variant was reported as a founder variant and accounted for >94% of the pathogenic variants underlying HMGCLD in Saudi Arabia (Mitchell et al., 1998; Al-Sayed et al., 2006; Pie et al., 2007). Furthermore, we identified two previously reported variants (c.206_207del; p(Ser69Cysfs*11), c.914_915delTT; p(Phe305Tyrfs*10)) in additional seven affected individuals (Mitchell et al., 1998; Al-Sayed et al., 2006; Menao et al., 2009; Alfadhel et al., 2016).

To reach the diagnosis, some accumulated toxic metabolites should be tested in urine as the disease is considered one of the organic academic disorders. Several studies demonstrated that the patient excreted abnormal amounts of leucine catabolites such as 3-methyl glutaconic, 3-hydroxy-3-methyl glutaric, 3-methyl glutaric, 3-hydroxy isovaleric, and glutaric acids in the urine, which were found in all our patients, and we believe it is the leading cause of metabolic acidosis and the cause of an apparently secondary carnitine deficiency (Alfadhel et al., 2017).

4In conclusion, our data demonstrate that HMGCLD is a pan-ethnic disease, although some variants are clustered in some geographic regions with close connections throughout history. Our study also provides the most extensive genotype analysis of HMGCLD patients from Saudi Arabia. In addition, we conclude that HMGCLD is a heterogeneous disease with a variable clinical presentation and outcome; thus, early detection of the disease contributes to proper intervention, minimizing recurrent metabolic decompensation, and therefore, a normal cognitive outcome. HMGCLD patients suffer from avoidable neurologically devastating metabolic decompensations, and based on our results, we recommend newborn screening (NBS) of Saudi ethnicity for the *HMGCL* [p.(Arg41Gln)] variant.

### Limitation

Such studies have limitations such as being prone to selection and information biases, unavailability of sufficient cases/data, and unavailability of the measurement of all the potential risk factors. However, such studies have the advantages of being quick, easy to perform, and helpful in addressing diseases of low incidence.

## Data Availability

The datasets presented in this study can be found in online repositories. The name of the repository, accession numbers, and link to the data can be found below: Leiden Open Variation Database (LOVD); Individual IDs: 00405120 - 00405175; https://databases.lovd.nl/shared/diseases/01895.
